# Supplementary figures and images for: The quality and complexity of pairwise maximum entropy models for large cortical populations
Source: PLoS Comput Biol. 2024 May 2;20(5):e1012074. doi: 10.1371/journal.pcbi.1012074 (PMC11093338; doi:10.1371/journal.pcbi.1012074)

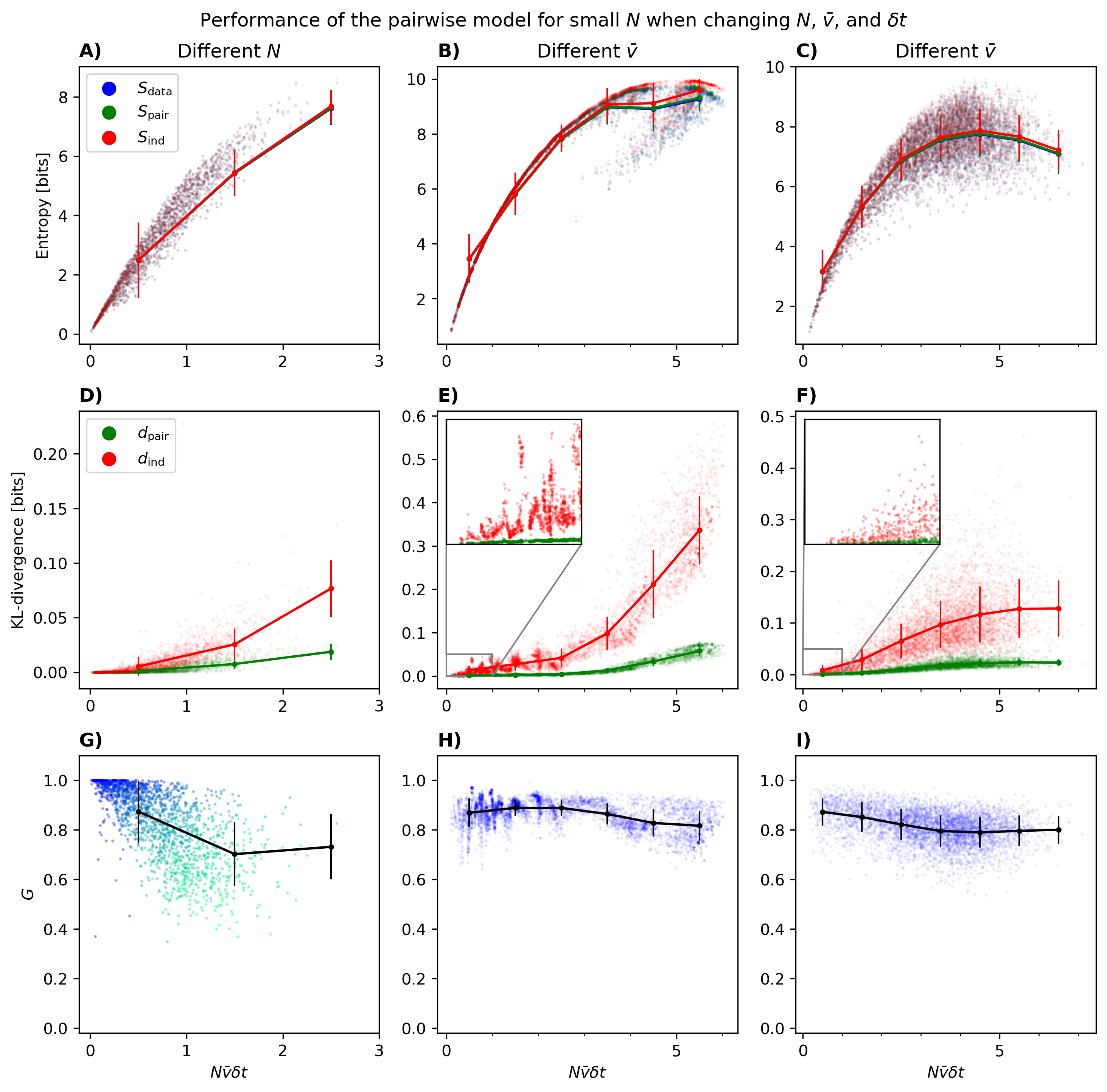

Supplement: S1 Fig — Everything is the same as in Fig 2, except that δt = 0.01 in the first column and N = 10 in the second and third column. (PNG) [file pcbi.1012074.s001.png]

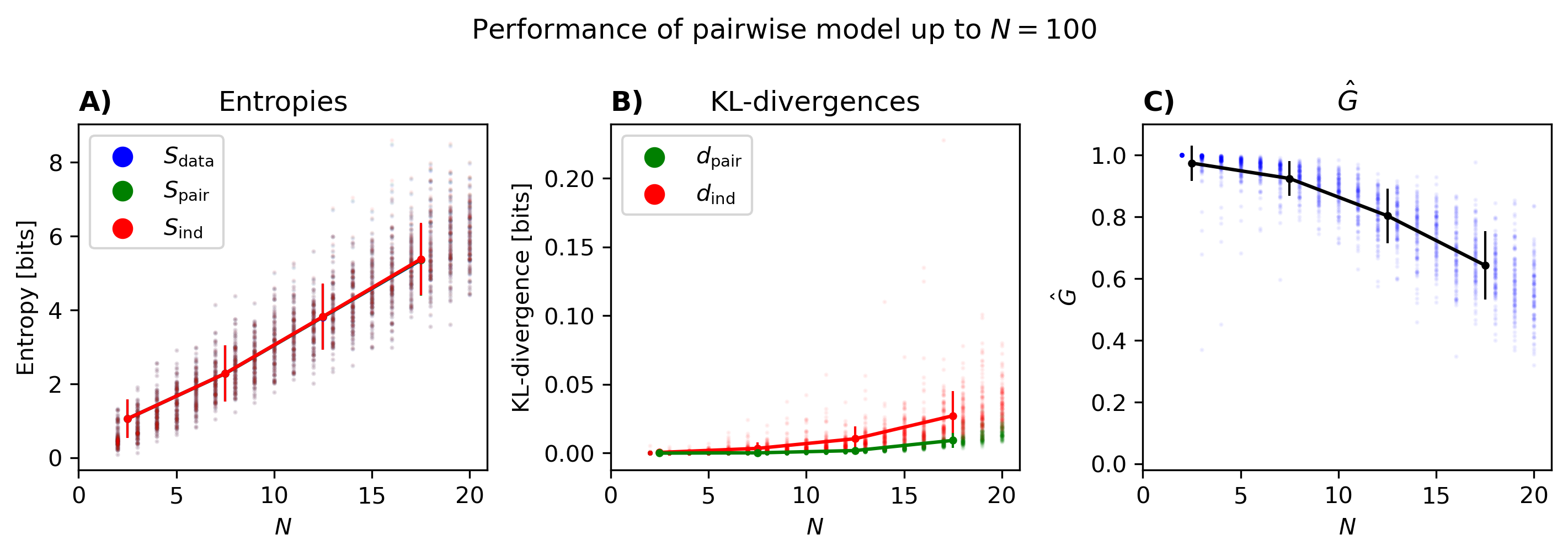

Supplement: S2 Fig — The first column in S1 Fig, except that Nν¯δt on the x-axis is replaced by N. (PNG) [file pcbi.1012074.s002.png]

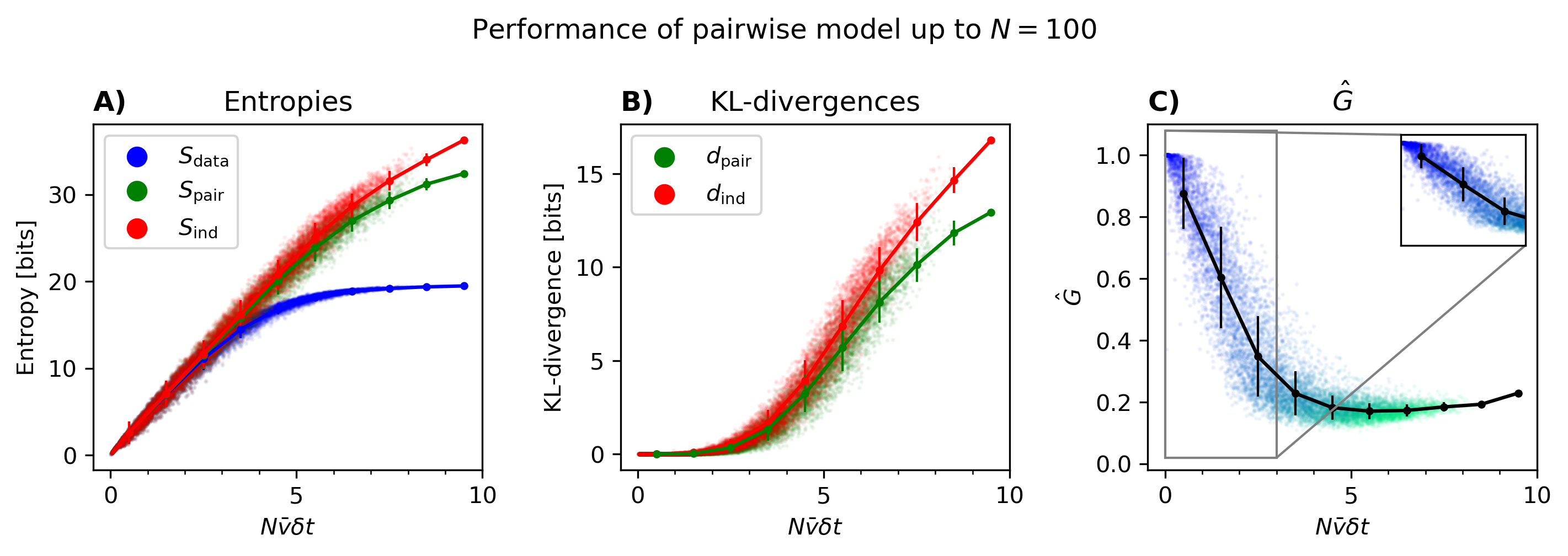

Supplement: S3 Fig — Everything is the same as in Fig 6, except for δt = 0.01. (PNG) [file pcbi.1012074.s003.png]

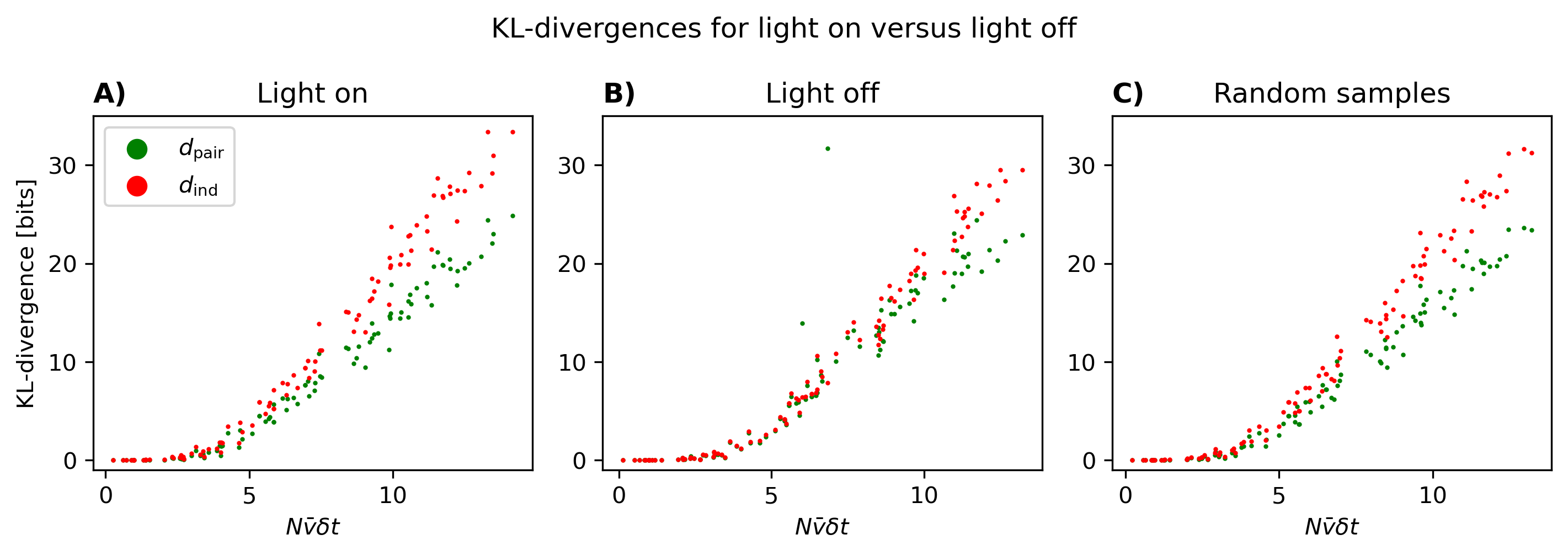

Supplement: S4 Fig — Everything is the same as in Fig 6, except for δt = 0.01. (PNG) [file pcbi.1012074.s004.png]

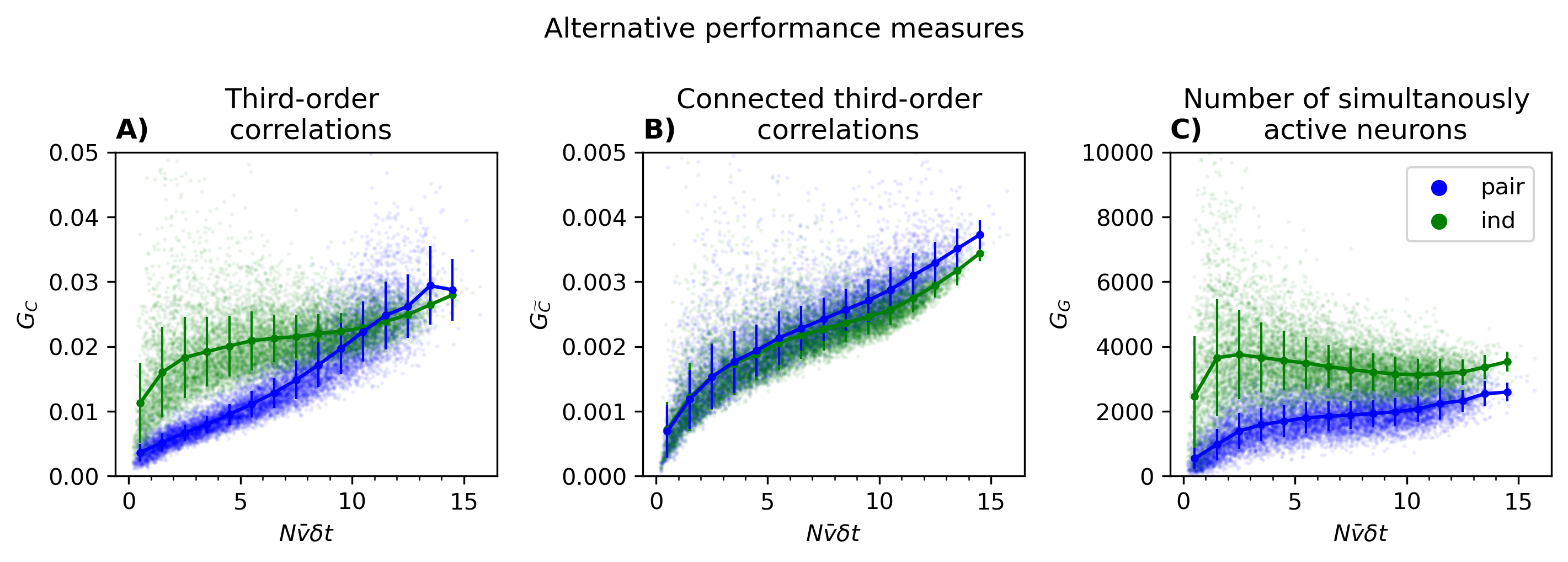

Supplement: S5 Fig — Root mean squared error of Cijk (A), C˜ijk (B), and Hm (C) between the data and the pairwise model (blue) and between the data and the independent model (green). This is an alternative visualization of the data presented in Fig 8. (PNG) [file pcbi.1012074.s005.png]

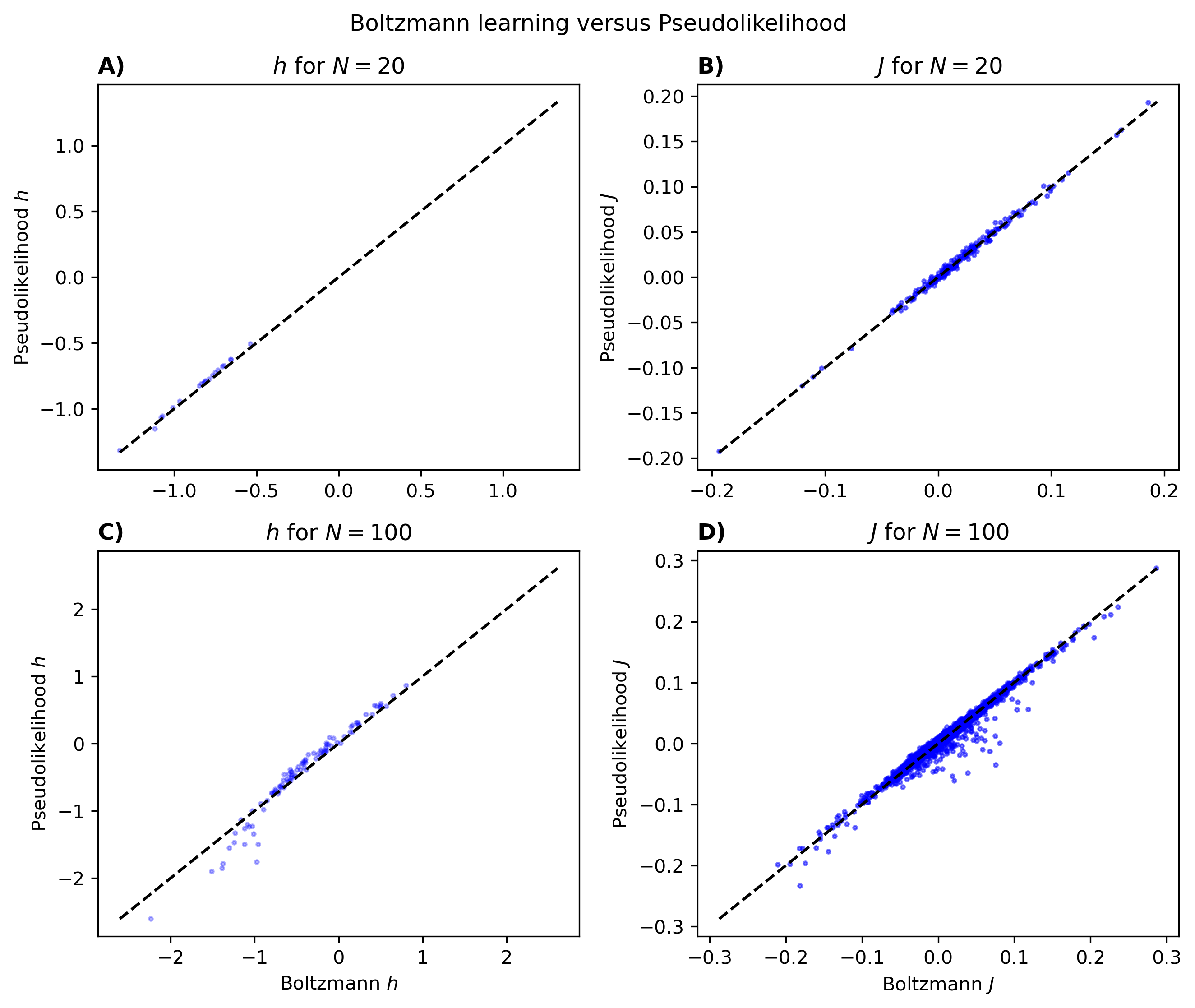

Supplement: S6 Fig — (A-B) A random subpopulation of 20 out of the 495 neurons were chosen. The pseudolikelihood parameters hiPL and JijPL were plotted against the Boltzmann learning parameters hiboltz and Jijboltz, which used a learning rate of η = 0.01, 40000 iterations, and 10000 samples per iteration. (C-D) A random subpopulation of 100 out of the 495 neurons were chosen. The pseudolikelihood parameters hiPL and JijPL were plotted against the Boltzmann learning parameters hiboltz and Jijboltz, which used a learning rate of η = 0.01, 80000 iterations, and 50000 samples per iteration. Further comparisons are available in [67]. (PNG) [file pcbi.1012074.s006.png]
