# Supplementary material for: The quality and complexity of pairwise maximum entropy models for large cortical populations
Source: PLoS Comput Biol. 2024 May 2;20(5):e1012074. doi: 10.1371/journal.pcbi.1012074 (PMC11093338; doi:10.1371/journal.pcbi.1012074)
Supplement: S1 Appendix — (PDF) [file pcbi.1012074.s007.pdf]

S1 Appendix In this section, we will outline some properties of the estimator described in Eq. (14). The starting point is to note that the summation of $\sum_{\hat{\mathbf{s}}\in\mathcal{O}}$ over the unique observed states in the numerator and the denominator is equivalent to

$$\sum_{\hat{\mathbf{s}}\in\mathcal{O}} \equiv \sum_{\hat{\mathbf{s}}\in\mathcal{D}} M(\hat{\mathbf{s}})^{-1} \tag{1}$$

where the summation is now over all samples in the data $\mathcal{D}$, $M(\mathbf{s})$ is the number of times state $\mathbf{s}$ is observed in $\mathcal{D}$ and M is the size of $\mathcal{D}$. With this notation, we have $p_{\text{data}}(\mathbf{s}) = \frac{M(\mathbf{s})}{M}$, and we can write

$$\hat{Z} = \frac{1}{M}\sum_{\hat{\mathbf{s}}\in\mathcal{D}} \frac{\exp[-E_{\text{pair}}(\hat{\mathbf{s}})]}{p_{\text{data}}(\hat{\mathbf{s}})} q(\hat{\mathbf{s}}) \tag{2}$$

where $E_{\text{pair}}(\mathbf{s}) = -\sum_i h_i s_i - \sum_{i<j} J_{ij} s_i s_j$ is the energy of the state $\mathbf{s}$ according to the pairwise model, and

$$q(\mathbf{s}) \equiv \frac{\exp[-E_{\text{pair}}(\mathbf{s})]}{\sum_{\mathbf{s}'} p_{\text{data}}(\mathbf{s}')\exp[-E_{\text{pair}}(\mathbf{s}')]}. \tag{3}$$

Note that the summation in the denominator of Eq. (3) can be taken over all states or only states that have been observed in the sample, that is, the states $\mathbf{s}$ where $p_{\text{data}}(\mathbf{s}) \neq 0$. For states in the latter group, namely those that actually occur in the sample and thus the sum in Eq. (2), we can write

$$q(\hat{\mathbf{s}}) = \left\{ p_{\text{data}}(\hat{\mathbf{s}}) \sum_{\hat{\mathbf{s}}'} \exp[-\Delta E_{\text{pair}}(\hat{\mathbf{s}}', \hat{\mathbf{s}}) - \Delta E_{\text{data}}(\hat{\mathbf{s}}', \hat{\mathbf{s}})] \right\}^{-1} \tag{4}$$

where

$$\Delta E_{\text{pair}}(\hat{\mathbf{s}}', \hat{\mathbf{s}}) = E_{\text{pair}}(\hat{\mathbf{s}}') - E_{\text{pair}}(\hat{\mathbf{s}}) \tag{5}$$

$$\Delta E_{\text{data}}(\hat{\mathbf{s}}', \hat{\mathbf{s}}) = -\log p_{\text{data}}(\hat{\mathbf{s}}') + \log p_{\text{data}}(\hat{\mathbf{s}}) \tag{6}$$

are the energy gap between $\hat{\mathbf{s}}'$ and $\hat{\mathbf{s}}$ according to the pairwise model and the data, respectively.

The first interesting thing to notice is that Eq. (2) has the familiar form of an importance sampling approximation to the partition function but with two crucial differences. First, the tractable sampling distribution that appear in the denominator in importance sampling is replaced by p_{data}. This means that we do not need to generate samples and directly employ the data to which the model is fit. Second, the samples are weighted by the factor $q(\mathbf{s})$ which is equal to one in importance sampling.

To better understand the effect of this weighting work, it is instructive to consider an example that reflects what is likely observed in the neural data. Suppose when states are ranked in decreasing order of probabilities according to the pairwise model and the data probabilities, the rankings $r(\mathbf{s})$ are the same and the probabilities exhibit Zipfian laws [1, 2]:

$$p_{\text{pair}}(\mathbf{s}) \propto r(\mathbf{s})^{-\eta_{\text{pair}}} \tag{7}$$

$$p_{\text{data}}(\mathbf{s}) \propto r(\mathbf{s})^{-\eta_{\text{data}}}, \tag{8}$$

which implies that

$$\exp[-\Delta E_{\text{pair}}(\mathbf{s}', \mathbf{s}) - \Delta E_{\text{data}}(\mathbf{s}', \mathbf{s})] = (r(\mathbf{s})/r(\mathbf{s}'))^{\eta_{\text{pair}}+\eta_{\text{data}}}. \tag{9}$$

In such cases the partition functions are easy to compute, that is $Z_{\text{pair}} = \zeta(\eta_{\text{pair}})$ and $Z_{\text{data}} = \zeta(\eta_{\text{data}})$, where $\zeta(\cdot)$ is the Riemann zeta function. But, as we show below,

considering how the weighting and approximation in Eq. (14) work in this case provides good insight into the operations at work.

Inserting Eq. (9) in Eq. (4), we have

$$q(\hat{\mathbf{s}}) = \left[p_{\text{data}}(\hat{\mathbf{s}}) r(\hat{\mathbf{s}})^{\eta_{\text{pair}}+\eta_{\text{data}}} \zeta(\eta_{\text{pair}} + \eta_{\text{data}})\right]^{-1} = \frac{\zeta(\eta_{\text{data}})}{\zeta(\eta_{\text{pair}} + \eta_{\text{data}})} r(\hat{\mathbf{s}})^{-\eta_{\text{pair}}}. \quad (10)$$

This shows that states in the data that are assigned a higher probability (lower rank) in the pairwise model are assigned a higher weight in Eq. (2) compared to standard importance sampling.

We can also use Eq. (10) to better understand why Eq. (14) performs better than approximating the partition function by $\exp(-E_{\text{pair}}[\mathbf{s}_0])/p_{\text{data}}(\mathbf{s}_0)$ or the average/median of $\exp(-E_{\text{pair}}[\mathbf{s}])/p_{\text{data}}(\mathbf{s})$ for the states observed in the sample (Fig. 4). Using Eq. (10), we can write

$$\hat{Z} = \sum_{\hat{\mathbf{s}} \in \mathcal{O}} \frac{\exp[-E_{\text{pair}}(\hat{\mathbf{s}})]}{p_{\text{data}}(\hat{\mathbf{s}})} [r(\hat{\mathbf{s}})^{\eta_{\text{pair}}+\eta_{\text{data}}} \zeta(\eta_{\text{pair}} + \eta_{\text{data}})]^{-1} \quad (11)$$

where the summation is over all states actually observed in the data. Here again, we obtain a weighted average of the ratios $\exp[-E_{\text{pair}}(\mathbf{s})]/p_{\text{data}}(\mathbf{s})$, with states with higher rank (lower empirical probability) assigned lower weights. This makes sense, since states with lower values of p_{data} are likely to deviate more from the corresponding p_{pair} leading to ratios that deviate more from Z_{ratio} than those with larger p_{data}, in particular the most observed state.

In the main text we compared $\hat{Z}$ to estimators based various combinations of the ratios $\exp[-E_{\text{pair}}(\mathbf{s})]/p_{\text{data}}(\mathbf{s})$ (see Fig 14). Given the similarity of our approximation to importance sampling, as noted above, in the following figure, we also compare $\hat{Z}$ with estimators based on importance sampling and reverse importance sampling [3]. We do this both for examples with $N = 100$ where we can compute the partition function exactly and also for experimentally recorded neural data of this size.

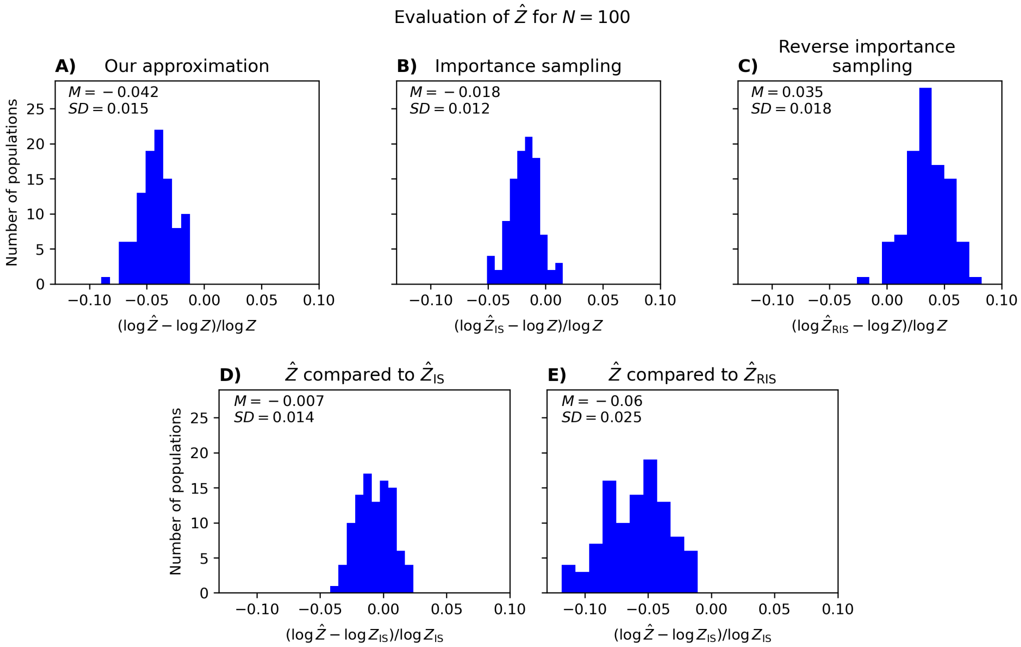

Evaluation of $\hat{Z}$ for large N. In panel A-C we construct PME models consisting of 20 independent groups of five spins ($N = 100$). The parameters h_i and (non-zero) J_{ij} were Gaussian with a mean of -1 and 0, respectively, and had a standard deviation of 0.5. We now have parameters for pairwise models, with $N = 100$ and consisting of independent groups of five spins, for which we can calculate Z exactly. In panel A, we then compare our approximation in Eq. (14), based on $T = 400000$ samples from the constructed pairwise model, to the exact Z. Similarly, we compare approximations based on importance sampling $\hat{Z}_{\text{IS}}$ (B) and reverse importance sampling $\hat{Z}_{\text{RIS}}$ [3] to the exact Z of the constructed pairwise model. In D-E we instead pick 100 random neural populations of $N = 100$ neurons and compare our approximation $\hat{Z}$ to the approximation based on importance sampling $\hat{Z}_{\text{IS}}$ (D) and reverse importance sampling $\hat{Z}_{\text{RIS}}$ (E).

References

1. Mora T, Bialek W. Are biological systems poised at criticality? Journal of Statistical Physics. 2011;144:268–302.

2. Tyrcha J, Roudi Y, Marsili M, Hertz J. The effect of nonstationarity on models inferred from neural data. Journal of Statistical Mechanics: Theory and Experiment. 2013;2013(03):P03005.

3. Liu Q, Peng J, Ihler A, Fisher III J. Estimating the partition function by discriminance sampling. In: Proceedings of the Thirty-First Conference on Uncertainty in Artificial Intelligence; 2015. p. 514–522.
