# Supplementary material for: The quality and complexity of pairwise maximum entropy models for large cortical populations
Source: PLoS Comput Biol. 2024 May 2;20(5):e1012074. doi: 10.1371/journal.pcbi.1012074 (PMC11093338; doi:10.1371/journal.pcbi.1012074)
Supplement: S2 Appendix — (PDF) [file pcbi.1012074.s008.pdf]

S2 Appendix When analyzing the performance of the PME model as measured by G, we have not included sampling biases that arise from our assumption that $p_{\text{true}} = p_{\text{data}}$ [1]. Here, we study to what degree our estimation of G and its dependence on $N\bar{\nu}\delta t$ change if we correct for such biases [2]. To correct the estimation of a quantity K, estimated from T samples, for finite sampling, we consider different proportions $r \in \{1/2, 1/5, 1/10, 1\}$ of the T samples. When $r < 1$, mutually exclusive proportions of the data were used to estimate K, and the mean $\bar{K}$ of these estimates was calculated. $\bar{K}$ was then fit to a second-order polynomial in $1/T$. We take the limit $T \to \infty$ which gives the corrected value of K. The results are shown below, where we can see that there are no discernible differences from the correction of sampling bias. We also estimated the entropy of the data used in the calculation of G using a Bayesian entropy estimator [3] and did not observe any substantial changes.

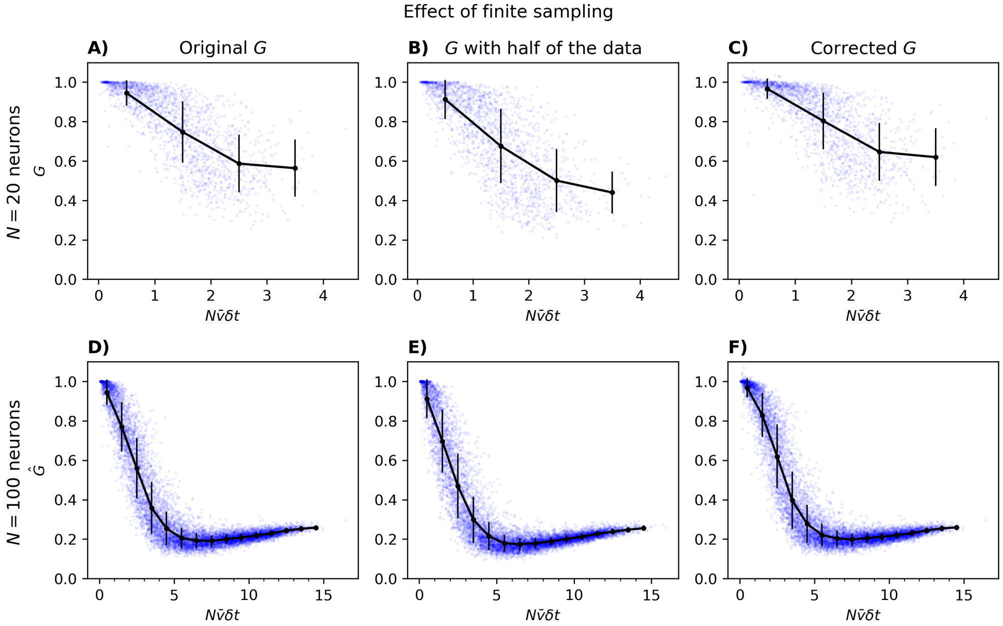

Finite sampling effects on G. (A-C) Same as in Fig 2A, but with only half the data and corrected for finite sampling [2]. (D-F) Same as in Fig 6, but with only half the data and corrected for finite sampling [2]. In panel B and E, a random collection of half of the samples was used for every population. This figure shows that the scaling of G and $\hat{G}$ is not extremely sensitive to the amount of data available.

References

1. Panzeri S, Senatore R, Montemurro MA, Petersen RS. Correcting for the sampling bias problem in spike train information measures. Journal of neurophysiology. 2007;98(3):1064–1072.

2. Strong SP, Koberle R, Van Steveninck RRDR, Bialek W. Entropy and information in neural spike trains. Physical review letters. 1998;80(1):197.

3. Archer EW, Park IM, Pillow JW. Bayesian entropy estimation for binary spike train data using parametric prior knowledge. Advances in neural information processing systems. 2013;26.
