# Supplementary material for: The quality and complexity of pairwise maximum entropy models for large cortical populations
Source: PLoS Comput Biol. 2024 May 2;20(5):e1012074. doi: 10.1371/journal.pcbi.1012074 (PMC11093338; doi:10.1371/journal.pcbi.1012074)
Supplement: S3 Appendix — (PDF) [file pcbi.1012074.s009.pdf]

S3 Appendix Here we report approximate equations that relate the statistics of the mean and pairwise correlations of the data to the mean and standard deviation of the couplings, as well as the main argument behind the condition of instability of the normal phase. These are largely classical results (see Ch. 3 of [1] and references therein), and we report them here for the sake of completeness.

Whether the SK model (Eq. (2) with $J_{ij} \sim \mathcal{N}(J_0/N, J_1^2/N)$ is its complex phase depends on the strength of the couplings and consequently on the strength and correlations between pairwise correlations. This can be determined using the $N \to \infty$ limit of the spin-glass susceptibility.

$$\mathcal{X}_{SG} = \frac{1}{N}\sum_{ij} C_{ij}C_{ji} = \frac{1}{N}\sum_{i\neq j} C_{ij}C_{ji} + \frac{1}{N}\sum_{i}(1-m_i^2) \tag{1}$$

Not that this is different from the normal susceptibility $\mathcal{X}_{ij} \equiv \partial m_i/\partial h_j = C_{ij}$ which is studied in transitions between different normal phases of the system. Intuitively, for weak couplings, pairwise correlations are weak (e.g. $\mathcal{O}(N^{-1})$), random and uncorrelated. Consequently, $\mathcal{X}_{SG}$ remains finite even for large N and the system is in its normal phase. As the couplings increase in strength, the correlation may be such that $\mathcal{X}_{SG}$ diverges for large N indicating the entrance into the complex phase [2]

The fluctuation-response relationship holds that

$$\mathcal{X}_{ij} \equiv \partial m_i/\partial h_j = C_{ij}, \tag{2}$$

where $\mathcal{X}_{ij}$ susceptibility. Applying this to the naive Mean-Field equation for m_i which is the same as Eq. (16) but without the second sum inside the hyperbolic tangent, we have

$$C_{ij} = (1-m_i^2)\left[\delta_{ij} + \sum_k J_{ik}C_{kj}\right]. \tag{3}$$

Summing both sides of Eq. (3) over $i, j, i \neq j$ and noting that $C_{kk} = (1-m_k^2)$, we get

$$\frac{J_0}{N} = \frac{\overline{C}}{(1-q)(1-q+N\overline{C})} \tag{4}$$

To derive an estimate of the standard deviation of the couplings, we again use the fluctuation-dissipation relationship, but this time applied to Eqs. 16. This yields

$$\mathcal{X}_{ij} = \mathbf{A}_{ij}^{-1} = C_{ij} \tag{5a}$$

$$A_{ij} = -J_{ij} - 2(J_{ij})^2 m_i m_j + \delta_{ij}\left[\sum_k J_{ij}^2(1-m_k^2) + \frac{1}{(1-m_i^2)}\right] \tag{5b}$$

Consequently,

$$\sum_{i,j} C_{ij}^2 = \sum_{i,j} C_{ij}C_{ji} = \sum_i \mathbf{A}_{ii}^{-2} = \mathrm{Tr}\mathbf{A}^{-2} \tag{6}$$

The left hand side of Eq. (6), divided by N is the spin-glass susceptibility, X_{SG}, and it can be show that as $N \to \infty$, for couplings scaled as the SK prescription, will be

$$\mathrm{Tr}\mathbf{A}^{-2} = \frac{NS}{1-J^2S}, \quad S = \frac{1}{N}\sum_i (1-m_i^2)^2 \tag{7}$$

nothing that $C_{ij} = 1 - m_i^2$, we can write for

$$\overline{C^2} \equiv \frac{1}{N(N-1)}\sum_{i\neq j} C_{ij}^2 = \frac{J^2S}{(N-1)(1-J^2S)} \tag{8}$$

which can be used to write

$$\frac{J_1^2}{N} = \frac{(N-1)\overline{C^2}}{NS(S+(N-1)\overline{C^2})} \tag{9}$$

The divergence of $N\overline{C^2}$, which implies the divergence of the spin-glass susceptibility $\mathcal{X}_{SG}$, gives the condition $J^2S = 1$ for the instability of the normal phase and the entrance to the complex phase [3].

To get an intuitive feeling of when and how the complexity is different in different phases of the model, one can consider the case of $J_1 = 0$ and $h_i = 0$, that is, when $J_{ij} = \frac{J_0}{N}$ which is simply the mean-field Ising model with the free energy as a function of the mean magnetization $m = m_i = \langle s_i \rangle$,

$$g(m) = -\frac{J_0 m^2}{2} + \frac{1}{2}[(1+m)\log[(1+m)/2] + (1-m)\log[(1-m)/2] \tag{10}$$

with the minima at the solutions

$$m = \tanh(J_0 m) \tag{11}$$

which is the same as Eq. (16), but with the second term inside the tanh being insignificant for large N. For $J < 1$, there is only one solution at $m = 0$, which is the non-magnetized paramagnetic phase. For $J > 1$ there are two solutions as the minima of the free energy, with $m \neq 0$; this is the magnetized ferromagnetic phase. Both phases are normal in the sense that the number of minima of the free energy does not increase with N. Making J_1 nonzero changes the story: the relevant equations become those of Eq. (16) known as the Thouless-Anderson-Palmet (TAP) equations [4], which can be shown to have exponentially many, in N, locally stable solutions. This situation arises because of the many spins with conflicting "interests" showing up due to the many positive and negative couplings between different pairs of spins, a situation that is not present for the mean-field Ising model.

References

1. Fischer KH, Hertz JA. Spin glasses. Cambridge university press; 1993.
2. Bray AJ, Moore MA. Evidence for massless modes in the'solvable model'of a spin glass. Journal of Physics C: Solid State Physics. 1979;12(11):L441.
3. de Almeida JR, Thouless DJ. Stability of the Sherrington-Kirkpatrick solution of a spin glass model. Journal of Physics A: Mathematical and General. 1978;11(5):983.
4. Thouless DJ, Anderson PW, Palmer RG. Solution of'solvable model of a spin glass'. Philosophical Magazine. 1977;35(3):593–601.
