# Supplementary material for: The quality and complexity of pairwise maximum entropy models for large cortical populations
Source: PLoS Comput Biol. 2024 May 2;20(5):e1012074. doi: 10.1371/journal.pcbi.1012074 (PMC11093338; doi:10.1371/journal.pcbi.1012074)
Supplement: Attachment — Submitted filename: report.pdf [file pcbi.1012074.s010.pdf]

In this work, V. L. K. Olsen, J. Whitlock and Y. Roudi study the performance of the pairwise maximum-entropy model (PME) to describe the spiking activity of large populations of neurons. The authors claim that the PME performs very well for small neural networks ($N \sim 5 - 10$ neurons), and that this good performance does not extend to larger neural populations ($N = 40$ or larger), and the authors' analysis suggests that the in these instances the PME model does not capture quite well the correlation structure of the data.

Maximum-entropy (ME) models have been widely misinterpreted and unproperly used in the past few years: as a result, a critical assessment of ME models such as the one proposed by the authors may be important for different scientific communities, and so is the general purpose and subject of this paper. In particular, I find interesting that the analysis provided by the authors may shed light on the regime where $N\bar{\nu}\delta t$ is not small which, according to the authors, has not been studied yet in the literature. However, the manuscript presents several issues, on both the scientific and presentational level, which need to be carefully addressed before the paper can be considered for publication in PLOS. Please find below list of the points that need to be addressed by the authors, divided into major and minor points.

Yours Sincerely,

Michele Castellana,
Physical Chemistry Curie,
Institut Curie,
Paris, France

Major issues:

- "In Fig 4, we evaluate these estimators by comparing them to the true value of Z for $N = 25$." The authors test their approximation for Z for $N = 25$, but then they use it for larger N. To make a convincing point, they should test the approximation for the values of N for which they actually use it, or at least for larger values of N, by comparing it, for instance, with Monte Carlo simulations.
- "similarity to Fig. 2G shows that using our approximation of Z leads to similar results as the exact enumeration, at least when we can perform this enumeration." See my comment above and revise accordingly.
- "In addition to the measures based on KL-divergences [...] predicted from the inferred pairwise model." This statement implies that the data used by the authors allows to compute from the data, not only second-, but also third-order correlations. As a result, the 'goodness' of the pairwise model should also discussed and assessed also by performing the inference with both second- and third-order correlations, and then quantifying how the resulting maximum-entropy distribution differs from the one with pair correlations only.
- "or approximate methods such as pseudo-likelihood maximization" Whenever they mention or use an approximation throughout the manuscript, the authors should carefully discuss both its benefits and limitations, and provide a short review of the cases where such approximation works and fails.
- "Of course, infinite data is unavailable, so p_{true} is often taken to be the frequency of each activity pattern s in the data, denoted by p_{data}." Again, the authors should discuss thoroughly the potential consequences of this approximation.
- "the performance of the pairwise model is not good because the independent model is already a pretty accurate description of the data". Given that the authors state that the performance of

the pairwise model is not good because the independent model is already accurate, this check should be made for the three-body-interaction model vs. the pairwise model, by including in the manuscript a maxium-entropy analysis with three-point correlations, see my comment above.

- “we will discuss the properties of this estimator in more detail in a future publication.” The authours should at least outline the procedure here.
- “The interpretation we prefer for the increase [...]”. Please provide an argument rather than a preference.

Minor issues:

- “the Ising model with random connectivity, that is, the Sherrington-Kirkpatrick (SK) model." The SK model is not an Ising model with random connectivity, please check the literature and rephrase.
- “the pairwise fitting procedure itself faces problems” Please explain.
- “We also plot the entropies, KL-divergences and the performance measure G, as a function of the”. “as a function” should be replaced by “as functions.”
- “In the previous sections, we performed the analyses without taking into account the area from which the neurons are recorded.” This should have been stressed at the beginning of the previous Sections.
- “This was interpreted as increasing the redundancy of the code.” This sentence is unclear, please rephrase.
- “from many conflicting couplings”: “conflicting” → “frustrated”.
