# Supplementary material for: The quality and complexity of pairwise maximum entropy models for large cortical populations
Source: PLoS Comput Biol. 2024 May 2;20(5):e1012074. doi: 10.1371/journal.pcbi.1012074 (PMC11093338; doi:10.1371/journal.pcbi.1012074)
Supplement: Attachment — Submitted filename: Response to Refereers PLoS CB.pdf [file pcbi.1012074.s011.pdf]

Dear Editor,

We would like to resubmit our manuscript “The quality and complexity of pairwise maximum entropy models for large cortical populations” to PLoS Computational Biology.

We are grateful to the reviewers for their detailed and constructive comments and we have addressed all their concerns in the revised version.

Below, we go through the reviewer’s comments point by point. We also provide our answers and reference the corresponding changes in the main text of the manuscript (in red). Following the comments from the reviewers, we have added several new sections, figures and appendices that we also point to in our responses below.

We believe our manuscript has substantially improved after the revision and trust that you will find it suitable for publication in PLoS Computational Biology.

Sincerely yours,
Yasser Roudi

Reviewer 1

1. Olsen et al fit a large number of Ising models to Neuropixel recordings from multiple cortical areas of feely moving rats from a public dataset. They analyze how the quality of the model degrades with different features of the data such as population size, time binning or firing rates. The paper seems technically sound, and rigorous in varying different aspects of the analysis pipeline, which empirically expand theoretical results from Ref.10. It also introduces a couple of interesting statistical elements, in particular a new Ising partition function estimator and some lessons on the complex relationship between different empirical metrics of model quality used in practice.

   We are very happy that the reviewer finds our results sound and rigorous and appreciates the statistical ideas we have introduced.

2. Experimentally we are not learning anything fundamentally new:

   We agree that the focus of our writing in the first version was more on model evaluation and not a new biological result. In the revised manuscript we have addressed this issue in 3 ways:

- We have employed PME models to assess the effect of sensory stimulation on higher-order correlations by comparing PME models in the auditory cortex during silence and during bursts of white noise. See line 478-484 and Fig. 11.
- We have also expanded on our results on the effect of light versus dark conditions in the visual cortex so that the effect of stimulation can be compared between auditory and visual cortex. See Fig. 11.
- As per the reviewer's suggestion (8) below, we have analyzed the structure of the inferred couplings within and between the visual and auditory cortices. See section "Couplings within and between areas", line 484-499 and Fig. 12 and 13.
-

We have also made changes to the Introduction and Discussion to reflect these additional points and results.

3. it is a much more precise characterization of the long known observation that a simple Ising model only accounts for data well in small populations. Still, the manuscript does the grunt work of checking how this conclusion could be affected by various data and analysis considerations.

Indeed, as we and others have argued in the past the pairwise models are likely to perform well in terms of KL-divergence only for small populations. However, as Reviewer 3 also notes, there still is a lot of misunderstandings about maximum entropy models. There are indeed recent claims such as maximum entropy "[...] approach has been successful for populations of N ~ 100 neurons" (https://arxiv.org/abs/2310.10860) and "These pairwise maximum entropy models have been strikingly successful in describing collective behavior not only in networks of real neurons […]" (https://arxiv.org/abs/2402.00007v1).

These statements and other similar ones in the literature are not completely incorrect, but imprecise and confusing because of the different ways different people have used and assessed the PME. We thus believe highlighting this issue and systematically evaluating different measures will be of great use to the community.

4. I find dubious the scientific relevance of fitting a single Ising model to data that combines several computationally distinct sensory/behavioral conditions from multiple brain areas, but that concern is somewhat outside the scope of this paper. Still, I wonder if lumping together multiple conditions in this way also affects analysis in ways that DO matter for the goal at hand: naively one would think that combining subsets of data with different statistics (due to different computational regimes) results in mixture distributions with more complex associated pairwise correlation structure. Please discuss.

We agree with the reviewer regarding combining data from different sensory/behavioural conditions and brain regions. For this reason, we had indeed confirmed that our results did not qualitatively change if we only included neurons from one area or experimental condition.

Following this comment, we now expanded on this issue (see Figure 9, 10, and 11). We also now performed more a more quantitative comparison between population recorded only from the visual and only from auditory cortices areas under different conditions. See response to point 2.

5. The explanation of the core metrics needs substantial improvement. In particular I get the moment matching argument for the entropy difference definition of the KL, but it is sloppily done; similarly, the form of the factorized model is not terribly intuitive or properly explained. I imagine that a reader not already quite familiar with MaxEnt models would have a hard time following that. Either provide references together with each equation to make clear where it's coming from, or (ideally and) explain it in an accessible way in the results.

Following this comment we have added further explanations to improve clarity and accessibility. In particular, we also spell out the steps in the moment matching argument. See line 183-187.

6. Similarly, while I appreciate the symmetry of having all quality measures ranging between 0 and 1 with zero 'as good as factorized model' and one 'as good as actual data', this involves additional nonstandard steps for some of the metrics and those need a little justification: why sqrt(l2 distance ratio) specifically? Please comment.

We agree that such definitions have a degree of arbitrariness to them. Since previous studies have only relied on visual inspection of the scatter plots of third-order interactions we attempted to perform quantitative comparisons in a simple way.

Following the reviewers comment, we have also included a figure showing the same data as figure 8 but now only looking at the root mean squared error without the normalizations in see Supplementary Figure S5.

7. What does “larger variability in firing rates” means precisely when discussing increases in G for the N<20 analysis? 1) the means of different neurons being more different, I.e. population heterogeneity, or 2) more variance in individual neural responses (which scales with the mean for both the factorized Bernoulli model or a more realistic Poisson firing)?

We apologize for the confusion. We meant the former and we have now clarified this on line 309.

8. Does the structure of the larger neural populations sub-factorize by area? If looking at the estimated Js in a mixes population including subsets of neurons from different brain regions then is that structure largely block diagonal, with weak across area interactions and stronger within area couplings?

We thank the reviewer for suggesting that we look into this.

We now test this in addition. In addition we also report results on how stable the stronger couplings are as more neurons are being added either from the same, or from a different area. Our results show that in general connections from within an area consistently have larger mean than those from outside the area. See the new section “Couplings within and between areas” and Fig. 12 and 13.

9. since the partition function estimator is critical for a good part of the results, its construction needs to be explained more clearly. I also don’t understand the logic of not describing its properties in the paper itself in a self-contained way.

We avoided this as we thought the paper would get too long. But since Reviewer 1 and Review 3 both request this, we have now added further explanations and evaluations to the current manuscript. See line 386-393 in the main text and the new appendix Appendix S7.

10. Critically, the results text is missing a clear explanation of how the entropy of the real data is being estimated for N>20; also how does one practically go from an estimate of Z to that for S_pair?

We have now clarified this but adding new text explaining this in lines 362-367 and lines 183-187.

11. Overall, across all analyses the critical variable affecting model quality is the product N\bar\nu\delta t. The fundamental message of all analyses is to argue for a decrease in model performance as a function of this quantity. Given this agenda, one would need a little more initial motivation to justify why is this the critical quantity in the original theory and why should one expect it to be meaningful outside the perturbation regime of the original theory.

    Following the reviewer's suggestion, we now provide a more intuitive explanation of where N\bar\nu\delta appears in the theoretical analysis and why it is a critical parameter in the study of PME models applied to neural data. See line 234-239 and 245-249.

12. I personally don't find the SK subsection of the Results useful or experimentally relevant , despite the precedent of similar analyses in the retina. I also fundamentally question the interpretation of such metrics given that the data distribution was obtained by marginalizing across experimental conditions.

    We have indeed tested this analysis on subsets of data from lights-on and lights-off conditions obtaining similar results.

    As we emphasize, and perhaps for a similar reason as the reviewer, we do not use an analogy with the SK to imply something about the neural code per se as done in the retina. We simply employ known results about the SK model to quantify how hard sampling from the fitted model is. We believe for this purpose, using such known results are quite suitable. This is now further emphaszied in lines 644-654. We also have limited our reference to the discussion of this results in the Discussion.

Minor:

13. Section … is mentioned in the 2nd paragraph go Methods/Dataset , probably a legacy of a previous numbered section version of the text

    - Thank you, this is now fixed.

14. When introducing the constraints on the first and second moments (eq 1a,b) they are referred to as mean and correlations which is strictly speaking a little misleading.

Unfortunately we are not quite sure how this can be misleading as the quantities are clearly defined and are commonly used in the literature. We apologize for this and would be happy to change our notation if the reviewer suggests a more clear notation.

15. Title in Fig 2C is wrong, should be Different delta t

    Thank you, this is now fixed.

16. Caption fig 3: nu bar typo

    Thank you, this is now fixed.

17. Also not sure what the color is supposed to mean in 2G, and both panels in figure 3. All need meaningful color legends

    We have expanded on the information about the color gradients in the figure text. See Fig. 2 (page 9) and Fig. 3 (page 10) of the current version.

18. DATA and CODE sharing: the data is public but the code for the results it is not , at least as far as i can see.

    The code is now available here:
    https://osf.io/dajc6/?view_only=9862613238544ad4b8b85b1b42451c30

Reviewer 2

1. The manuscript describes quantification of maximum entropy models to fit statistics of recorded networks in the brain of a foraging rat. The recorded cells were distributed in several brain regions including visual, auditory, motor and somatosensory cortices. The authors perform analysis of the success of the PME models to predict several other statistics of networks' dynamics. Overall, the analysis looks good and well performed.

   We are pleased that the reviewer finds our analysis well-performed.

2. The authors also analyze predictions to larger populations and claim that the PME failed to predict the activity structure of these networks, a well know fact.

   There are still numerous papers that considers PME's as an excellent model for neural data. Two recent examples are "[…] this approach has been successful for populations of N ~ 100 neurons" (https://arxiv.org/abs/2310.10860) and "These pairwise maximum entropy models have been strikingly successful in describing collective behavior not only in networks of real neurons […]" (https://arxiv.org/abs/2402.00007v1).

As Reviewer 3 also states, there are still large misunderstandings about the PME models. A part of this emanates from the different ways different people have used and assessed the PME models. Indeed, in addition to showing the failure at large N when measured via KL-divergence we also show that pairwise models may perform quite well in predicting third-order correlations or probability of simultaneous spikes, in the regime where they fail in terms of KL-divergence. PME models, and indeed its failures, can also be used as a tool to assess the importance of higher-order correlations.

Given the above and as suggested by reviewer 1, we have further looked into how pairwise models perform between different stimulation conditions and across brain regions and have also studied the functional connections that emerge. We would also note that we show how the performance of the PME can be positively/negatively affected by the choice of time bin and average firing rate of the neurons.

We have now changed the main text to reflect the new results, and also changed the introduction and discussion so that the focus is not primarily on the failures of the PME.

Reviewer 3

In this work, V. L. K. Olsen, J. Whitlock and Y. Roudi study the performance of the pairwise maximum- entropy model (PME) to describe the spiking activity of large populations of neurons. The authors claim that the PME performs very well for small neural networks (*N* 5 10 neurons), and that this good performance does not extend to larger neural populations (*N* = 40 or larger), and the authors' analysis suggests that the in these instances thePME model does not capture quite well the correlation structure of the data.

1. Maximum-entropy (ME) models have been widely misinterpreted and unproperly used in the past few years: as a result, a critical assessment of ME models such as the one proposed by the authors may be important for different scientific communities, and so is the general purpose and subject of this paper. In particular, I find interesting that the analysis provided by the authors may shed light on the regime where *Nv6t* is not small which, according to the authors, has not been studied yet in the literature.

We are happy that the reviewer is finding our analysis interesting. We also agree with the reviewer that there is a lot of confusion regarding the interpretation of maximum entropy models and we believe our work helps the community in this respect.

2. However, the manuscript presents several issues, on both the scientific and presentational level, which need to be carefully addressed before the paper can be considered for publication in PLOS. Please find below list of the points that need to be addressed by the authors, divided into major and minor points.

We are most grateful for your detailed comments. In what follows we provide a point-by-point response indicating where we have made the necessary changes to address them.

Yours Sincerely,
Michele Castellana,
Physical Chemistry Curie,
Institut Curie,
Paris, France

Major issues:

3. "In Fig 4, we evaluate these estimators by comparing them to the true value of Z for N = 25." The authors test their approximation for Z for N= 25, but then they use it for larger N. To make a convincing point, they should test the approximation for the values of N for which they actually use it, or at least for larger values of N , by comparing it, for instance, with Monte Carlo simulations.

Following this comment we have further tested our approximation of Z, showing

(1) that it is close to the true Z of simulated systems for which we could exactly calculate Z. This was done for networks of 100 spins consisting of 20 independent groups of five spins; similar analyses were performed for gasses of dimer (only connected paired spins) and triplets finding the same results.

(2) that it is close to Monte Carlo-based partition function estimators for neural populations of 100 neurons.

Furthermore, following your comment numbered 9 below and comment numbered 9 reviewer 1, we now provide more details about the nature of the estimator we used here and how it can be further analysed and advanced.

Both of these additions results can be found in Appendix 7.

4. “similarity to Fig. 2G shows that using our approximation of Z leads to similar results as the exact enumeration, at least when we can perform this enumeration.” See my comment above and revise accordingly.

We believe the new comparisons and explanations added in response to comment (3) above addressed this issue.

5. “In addition to the measures based on KL-divergences [...] predicted from the inferred pairwise model.” This statement implies that the data used by the authors allows to compute from the data, not only second-, but also third-order correlations. As a result, the ’goodness’ of the pairwise model should also discussed and assessed also by performing the inference with both second- and third-order correlations, andthen quantifying how the resulting maximum-entropy distribution differs from the one with pair correlations only.

Thanks for this suggestion. We now discuss the effect of adding third-order correlations to populations where the independent model is already good (small d_ind) and to populations where the independent model is insufficient (large d_ind). See line 329-347.

6. “or approximate methods such as pseudo-likelihood maximization” Whenever they mention or use an approximation throughout the manuscript, the authors should carefully discuss both its benefits and limitations, and provide a short review of the cases where such approximation works and fails.

Following this comment we have now made the following changes

- We provide citations showing that pseudolikelihood maximization generally approximates exact Boltzmann learning well when the data length is large. See line 177-176.
- We also show new results confirming this in our data in one of the populations studied here. See Supplementary Figure 6. In the caption of this figure, we also

added citation to a thesis where comprehensive comparisons with other approximations are also performed.

- We show that our main results persist both when using Boltzmann learning (Figure 14) and when using other approximate methods (Figure 15).

7. “Of course, infinite data is unavailable, so p_{true} is often taken to be the frequency of each activity pattern s in the data, denoted by p_{data}.”Again, the authors should discuss thoroughly the potential consequences of this approximation.

In Appendix 8, we show that our results are very similar both when removing half of the data and when correcting for the finite sampling bias. This is also now emphasized in lines 255-256.

8. “the performance of the pairwise model is not good because the independent model is already a pretty accurate description of the data”.Given that the authors state that the performance of the pairwise model is not good because the independent model is already accurate, this check should be made for the three-body-interaction model vs. the pairwise model, by including in the manuscript a maxium-entropy analysis with three-point correlations, see my comment above.

Thanks! We have now added new analysis addressing this as described in our response to your comment numbered 5 above.

9. “we will discuss the properties of this estimator in more detail in a future publication.” The authours should at least outline the procedure here.

We have now added a new Appendix where we outline how the new estimator can be analyzed, its relations to importance sampling, and how the analysis can be extended. See Appendix 7.

10. “The interpretation we prefer for the increase [...]”. Please provide an argument rather than a preference.

- We have now re-written this part. See line 644-654.

Minor issues:

11. “the Ising model with random connectivity, that is, the Sherrington-Kirkpatrick (SK) model." The SK model is not an Ising model with random connectivity, please check the literature and rephrase.

We have now changed this sentence. See line 62-63.

12. “the pairwise fitting procedure itself faces problems” Please explain.

This is now removed and the text is re-written in particular following your comments regarding the third-order interactions.

13. “We also plot the entropies, KL-divergences and the performance measure G, as a function of the”. “as a function” should be replaced by“as functions.”

Thank you, this is now fixed.

14. “In the previous sections, we performed the analyses without taking into account the area from which the neurons are recorded.” Thisshould have been stressed at the beginning of the previous Sections.

Thanks! We now reiterated this issue at the start of the results section. See line 228-230 .

15. “This was interpreted as increasing the redundancy of the code.” This sentence is unclear, please rephrase.

We have now rephrased this sentence. See line 530-533.

16. “from many conflicting couplings”: “conflicting” → “frustrated”.

Thank you, this is now fixed.
